# SARS-CoV-2 infects cells lining the blood-retinal barrier and induces a hyperinflammatory immune response in the retina via systemic exposure

**DOI:** 10.1371/journal.ppat.1012156

**Published:** 2024-04-10

**Authors:** Monu Monu, Faraz Ahmad, Rachel M. Olson, Vaishnavi Balendiran, Pawan Kumar Singh

**Affiliations:** 1 Department of Ophthalmology, Mason Eye Institute, University of Missouri School of Medicine, Columbia, Missouri, United States of America; 2 Laboratory for Infectious Disease Research, University of Missouri, Columbia, Missouri, United States of America; 3 Department of Veterinary Pathobiology, University of Missouri, Columbia, Missouri, United States of America; University of Texas Medical Branch at Galveston, UNITED STATES

## Abstract

SARS-CoV-2 has been shown to cause wide-ranging ocular abnormalities and vision impairment in COVID-19 patients. However, there is limited understanding of SARS-CoV-2 in ocular transmission, tropism, and associated pathologies. The presence of viral RNA in corneal/conjunctival tissue and tears, along with the evidence of viral entry receptors on the ocular surface, has led to speculation that the eye may serve as a potential route of SARS-CoV-2 transmission. Here, we investigated the interaction of SARS-CoV-2 with cells lining the blood-retinal barrier (BRB) and the role of the eye in its transmission and tropism. The results from our study suggest that SARS-CoV-2 ocular exposure does not cause lung infection and moribund illness in K18-hACE2 mice despite the extended presence of viral remnants in various ocular tissues. In contrast, intranasal exposure not only resulted in SARS-CoV-2 spike (S) protein presence in different ocular tissues but also induces a hyperinflammatory immune response in the retina. Additionally, the long-term exposure to viral S-protein caused microaneurysm, retinal pigmented epithelium (RPE) mottling, retinal atrophy, and vein occlusion in mouse eyes. Notably, cells lining the BRB, the outer barrier, RPE, and the inner barrier, retinal vascular endothelium, were highly permissive to SARS-CoV-2 replication. Unexpectedly, primary human corneal epithelial cells were comparatively resistant to SARS-CoV-2 infection. The cells lining the BRB showed induced expression of viral entry receptors and increased susceptibility towards SARS-CoV-2-induced cell death. Furthermore, hyperglycemic conditions enhanced the viral entry receptor expression, infectivity, and susceptibility of SARS-CoV-2-induced cell death in the BRB cells, confirming the reported heightened pathological manifestations in comorbid populations. Collectively, our study provides the first evidence of SARS-CoV-2 ocular tropism via cells lining the BRB and that the virus can infect the retina via systemic permeation and induce retinal inflammation.

## Author summary

SARS-CoV-2 is known to cause several ocular manifestations in COVID-19 patients; however, the role of eyes in viral transmission and ocular tissue tropism remains elusive. The presence of viral remnants in various ocular tissues and fluids from COVID-19 patients has led to an assumption that SARS-CoV-2 may be transmitted through the eyes. Here, we show that SARS-CoV-2 ocular tropism is through cells lining the BRB. SARS-CoV-2 not only infects the various parts of the eye via systemic exposure but also induces a hyperinflammatory immune and antiviral response in the retina. Unexpectedly, the corneal epithelium was found to be resistant to SARS-CoV-2 infection, and ocular exposure of SARS-CoV-2 failed to cause lung pathology and moribund illness. Cells lining the BRB showed induced expression of viral entry receptors and enhanced susceptibility towards SARS-CoV-2-induced cell death, which is further potentiated with comorbidities such as hyperglycemia. Our findings from this study shed light on the role of BRB in SARS-CoV-2 ocular tropism and the role of eyes in viral transmission.

## Introduction

The global pandemic of COVID-19 caused by the extremely contagious SARS-CoV-2 affected nearly 772.83 million people and caused 6.98 million deaths worldwide as of December 27^th^, 2023 [1]. In addition to causing severe acute respiratory syndrome, COVID-19 has shown persistent ocular complications in human patients [2–5]. SARS-CoV-2 has demonstrated ocular tissue tropism with pervasive ocular manifestations, including conjunctivitis, keratoconjunctivitis, episcleritis, hyperemia, chemosis, epiphora, dry eye or foreign body sensation, eye redness, tearing, itching, ocular pain, cotton wool spots, hyperreflective lesions at the level of ganglion cell and inner plexiform layers, retinal artery and vein occlusion, retinal hemorrhage, vascular sheathing, macular neuroretinopathy, optic nerve infarction, optic nerve edema, optic neuropathies, cerebral vein thrombosis, uveitis, and glaucoma [2,6–15]. Recently, we reviewed the ocular complications caused by many conventional and emerging viruses, including SARS-CoV-2, which is considered relatively uncommon due to underreporting or improper diagnosis [16]. Early during the pandemic, studies on SARS-CoV-2 were primarily focused on respiratory manifestations of the virus with little or no knowledge of its ocular disease sequelae; however, more recent data have supported a much higher incidence of ocular comorbidities. The overall prevalence of ocular manifestations among COVID-19 patients has been shown to be 11.03% [17] and even higher (42.8%) amongst juveniles [18]. Although many studies have demonstrated the expression of SARS-CoV-2 binding receptors, angiotensin-converting enzyme 2 (ACE2) and a transmembrane serine protease, TMPRSS2, on ocular surfaces [19,20] and the presence of viral RNA in conjunctival swabs [21,22], tears [23,24], and aqueous humor [25], the possible ocular transmission, tropism, pathology, and long-term effect of COVID-19 on eye remains inconclusive. Moreover, patients with comorbidities such as diabetes have demonstrated increased SARS-CoV-2 morbidity and severity. COVID-19 has also been shown to induce hyperglycemia in patients without a history of diabetes [26]. However, how hyperglycemia modulates SARS-CoV-2 ocular transmission and tropism is unknown.

SARS-CoV-2 spreads through mucosal surfaces such as the nose, mouth, or eye exposed to infectious aerosols. Being an immune-privileged organ, the eye becomes a safe haven and potential site for viral replication. Several viruses, including Ebola, Zika, SARS-CoV, and MERS-CoV, can cause ocular complications associated with viral remnants in the eyes despite clearing from systemic circulation [27–31]. Anecdotal evidence has speculated that the ocular surface may serve as a route of SARS-CoV-2 transmission via the nasolacrimal duct [15,32–34]. The first posited case of ocular transmission of SARS-CoV-2 was the death of an ophthalmologist in Wuhan, China, who contracted COVID-19 while treating an asymptomatic glaucoma patient [32]. Since then, multiple clinical studies have demonstrated SARS-CoV-2 positivity in the eye. In addition to clinical studies, SARS-CoV-2 has been shown to infect human photoreceptor and ganglion cells [35], conjunctival epithelial cells [36], primary conjunctival, scleral, and limbal cells lining the corneal surface [37,38], and stem cells derived retinal organoids [35,37]. A recent preclinical study demonstrated ocular tropism of SARS-CoV-2 via neuronal invasion following intranasal exposure [39]. Despite many clinical and preclinical studies indicating the association of SARS-CoV-2 to ocular manifestation, a consensus on ocular tropism, whether the virus permeates eyes through ocular surface or through systemic exposure, is lacking. Thus, studies investigating the role of eye in SARS-CoV-2 ocular tropism, associated pathology, and its long-term consequences are of utmost importance in discovering preventive or therapeutic strategies.

In this study, we sought to determine the role of eyes in SARS-CoV-2 transmission and tropism using ocular and inhalation routes of exposure in K18-hACE2 mice. Our data show that the ocular tropism of SARS-CoV-2 is via cells lining the BRB, and intranasal exposure can cause a hyperinflammatory response in the retina along with mortality. Unexpectedly, we found primary human corneal cells and mouse cornea comparatively resistant to SARS-CoV-2 infection, and ocular surface exposure failed to cause viral dissemination and infection in the lungs. Furthermore, administration of recombinant spike protein caused sustained microaneurysm, retinal atrophy, RPE mottling, and vein occlusion in mouse eyes. SARS-CoV-2 elicited an innate immune response and induced cell death in BRB cells. Hyperglycemia augmented the BRB permissivity, viral entry receptors expression, and SARS-CoV-2-induced cell death in BRB cells. Collectively, our *in vivo* and *in vitro* findings provide novel insights into the pathogenesis of SARS-CoV-2 in the eye. To our knowledge, this study is the first to describe the role of cells lining the BRB in SARS-CoV-2 infectivity, ocular tropism, and associated pathology.

## Results

### Intranasal exposure of SARS-CoV-2 exhibited ocular infiltration, whereas ocular exposure failed to cause lung infection

**Fig 1:**
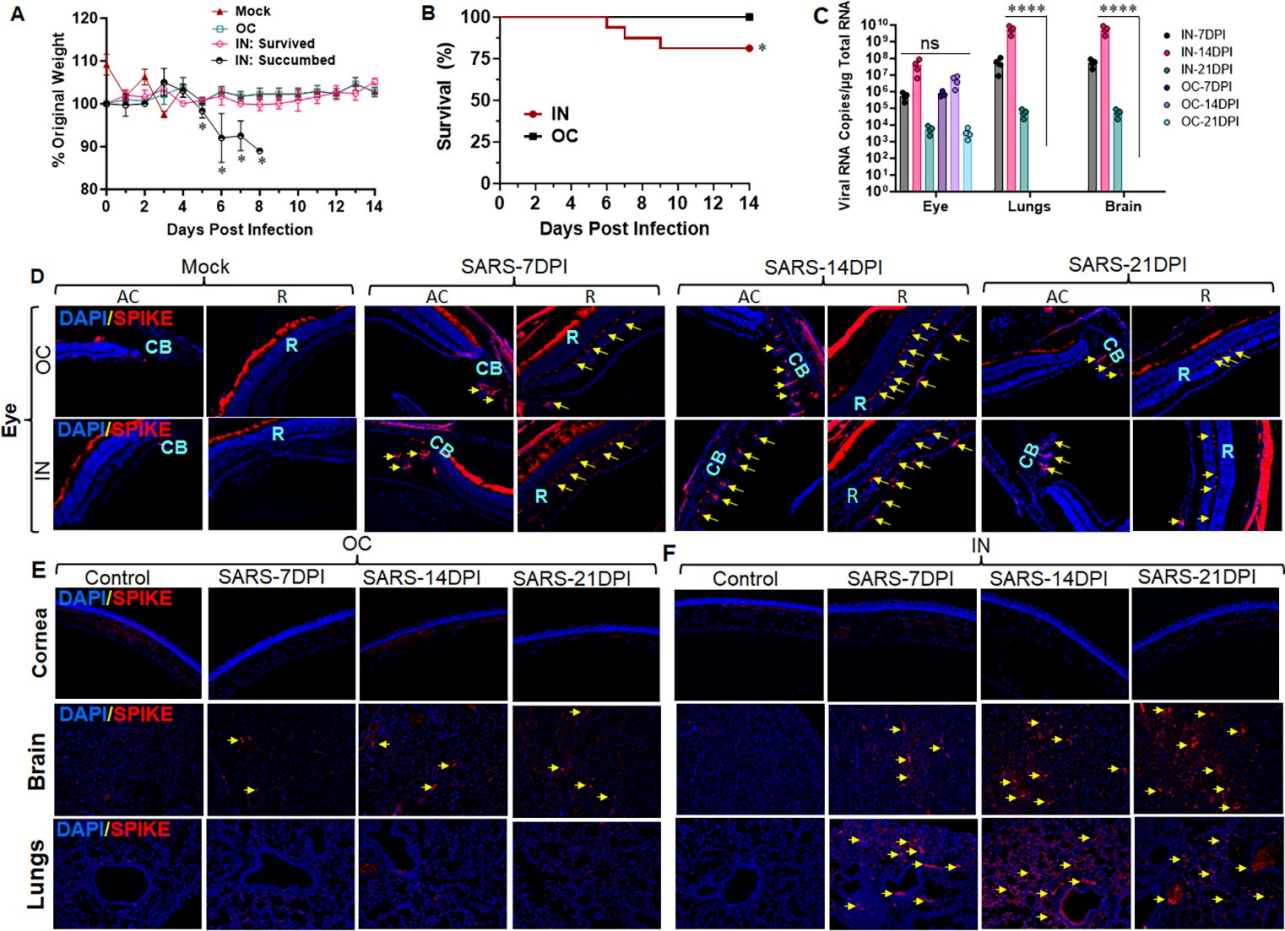
Intranasal exposure of SARS-CoV-2 exhibited ocular tropism to the eye, lungs, and brain, whereas ocular exposure failed to inseminate the virus to the lungs. K18-hACE2 mice (n = 4 per time point) were infected with SARS-CoV-2 strain USA-WA1/2020 (0.4x10^3^ PFUs) by Intranasal (IN) or Ocular (OC) route for 7, 14, and 21 days. **(A)** Body weight was recorded, and changes were shown as the percentage of starting body weight. **(B)** Survival was evaluated and indicated days post-infection (dpi). The IN-exposed group that showed weight loss and mortality was marked as succumbed and excluded from the downstream analysis. On the respective endpoints, the eyes, lungs, and brain tissue from the surviving animals were subjected to immunofluorescence staining for SARS-CoV-2 Spike protein (red) and DAPI nuclear stain (blue). The representative staining for viral Spike proteins is indicated with yellow arrows in **(D)** Eye globe (AC: Anterior chamber, R: Retina, CB: Ciliary bodies); **(E & F)** Cornea, Brain, and Lung tissue. Image magnification, X 200. **(C)** The Viral RNA levels in the eye globe, lungs, and brain were assessed using qPCR. Statistical significance between the experimental groups were determined using two-way ANOVA, Mock vs. IN-succumbed (A), and IN vs OC (C) and Kaplan-meier test (A). *P< 0.05, ****P<0.0001.

Several respiratory and neurotropic viruses, such as influenza, coronaviruses, Ebola, Zika, and West Nile viruses, have shown ocular tropism and caused ocular complications in humans [40,41]. Recent studies have demonstrated the presence of SARS-CoV-2 in various ocular tissues with or without ocular manifestations in COVID-19 patients [34,36]. However, it is unclear whether the ocular tropism of SARS-CoV-2 is via ocular surface exposure or systemic routes. It is also unknown whether ocular exposure can transmit the virus and cause lung infections. Therefore, in this study, we sought to determine the role of eye in SARS-CoV-2 tropism and transmission. We used a well-established human ACE2 (hACE2) expressing transgenic mice, where hACE2 is placed under keratin 18 (K18) promotor (K18-hACE2 mice). K18-hACE2 mice models are the most commonly used and reproducible model that has shown dose-dependent respiratory manifestations and lethality with SARS-CoV-2 infection [42]. In the current study, first, we titrated the infective dose by infecting K18-hACE2 mice with 0.4x10^3^, 0.4x10^4^, and 0.4x10^5^ PFUs of SARS-CoV-2 via intranasal (IN) route. We observed dose-dependent mortality in these animals with ~25% mortality by day 8 or 9 in 0.4x10^3^ PFU-infected mice, and therefore, we used this dose for the remainder of the study. Following dose titration, we challenged K18-hACE2 mice with 0.4x10^3^ PFU of SARS-CoV-2 via ocular (OC) and IN routes. We observed ~10–20% weight loss with ~25% mortality (succumbed) in IN-exposed mice by day 9, whereas the OC route did not cause weight loss or moribund illness (**Fig 1A and 1B**). We used the survived mice from the IN and OC groups for the downstream pathological evaluations. We assessed the presence of viral S-antigen in both IN- and OC-exposed mice in eye globes, lungs, and brains following 7-, 14-, and 21- days post-infection (DPI) using immunofluorescence (IFA) staining. Our data show the substantial presence of viral antigens in various parts of the eye at days 7 through 21, with a peak at day 14 post-infection in both IN and OC-exposed mice (**Fig 1D**). We detected viral antigens in both anterior and posterior segments of the eye, including retinal layers, iridocorneal section, and ciliary bodies in these mice (**Fig 1D**). However, we could not detect any viral remnants in the corneal tissue in either IN or OC-exposed groups (**Fig 1E and 1F**). In addition, IN exposure exhibited a substantial presence of viral S-antigen in the lung and brain in contrast to the OC route (**Fig 1E and 1F**). To further confirm the dissemination of SARS-CoV-2 to eye, lungs, and brain following IN and OC exposures, we measured the viral RNA (N-gene) copies using quantitative (q)PCR. Our data indicate the presence of viral RNA in the eye globes in both IN and OC-exposed groups; however, the lung and brain show the presence of viral RNA copies only with IN exposures (**Fig 1C**).

Together, these findings revealed that viral tropism in the eye, brain, and lungs is caused by systemic (IN) exposures. Moreover, the ocular surface exposure can establish ocular tissue tropism but does not disseminate the virus to distal organs such as the lungs and brain or cause moribund illness.

### Intranasal exposure of SARS-CoV-2 activated a hyperinflammatory immune response in the retina

**Fig 2:**
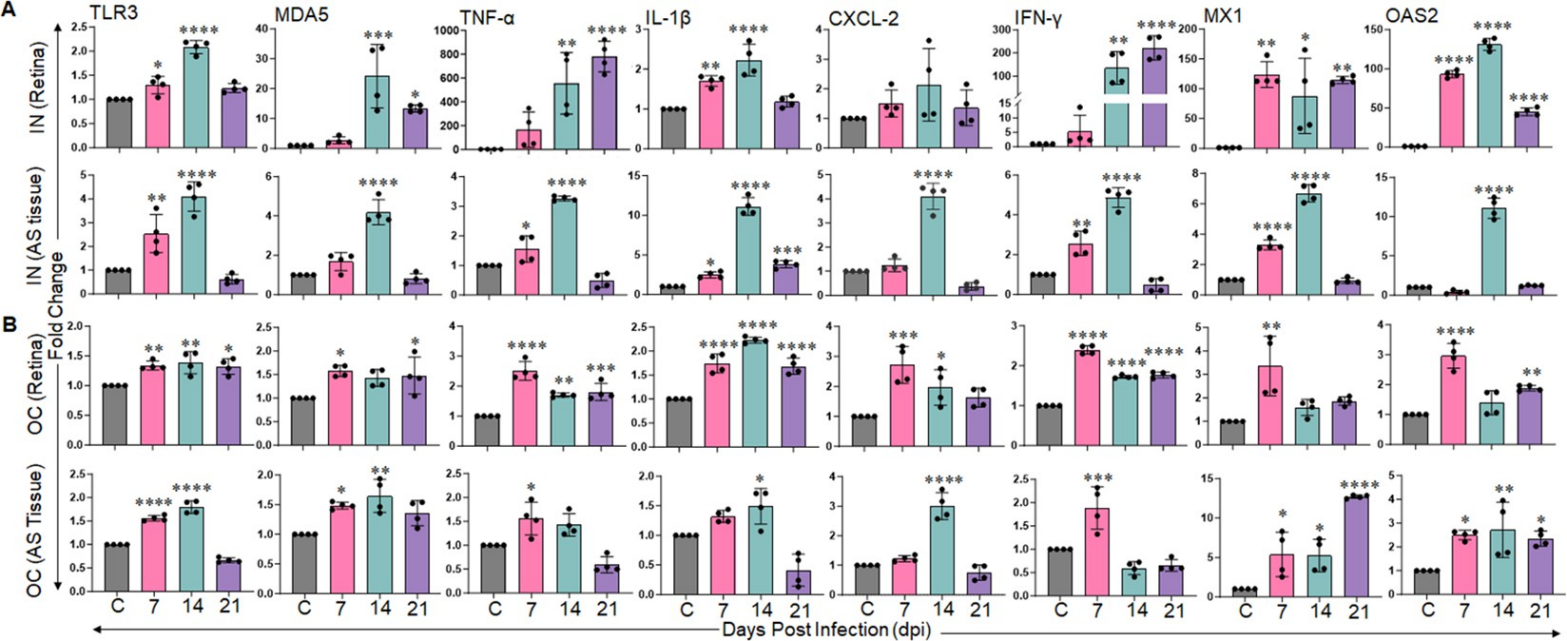
Intranasal exposure of SARS-CoV-2 elicits a hyperinflammatory and antiviral response in the retina. K18-hACE2 mice (n = 4/ time points) were challenged with SARS-CoV-2 strain USA-WA1/2020 (0.4x10^3^ PFUs) by **(A)** intranasal (IN) or **(B)** ocular (OC) route for 7, 14, and 21 days. At respective endpoints, anterior segment (AS) and retinal tissue were harvested and subjected to RNA extraction and qPCR for indicated genes. The bar graph represents means ± SD. Statistical differences between the experimental groups were determined using one-way ANOVA. *P< 0.05, **P<0.005, ***P<0.0005, ****P<0.0001.

Acute respiratory distress syndrome (ARDS) is one of the leading causes of death in COVID-19 patients, and is triggered by elevated levels of proinflammatory cytokines, often referred to as a cytokine storm. SARS-CoV-2 has also been shown to induce retinal inflammation in clinical and preclinical studies [39,43]. As SARS-CoV-2 was found to infect ocular tissue via both IN and OC routes in this study, we further examined the innate inflammatory response generated in the retina and anterior segment tissues from these infected mice. To assess the innate immune/inflammatory response, we measured the expression of pattern-recognition receptors (PRRs) [Toll-like receptor-3 (TLR3), Melanoma differentiation-associated protein-5 (MDA5)], inflammatory cytokines/chemokines [Tumour necrosis factor (TNF)-α, interleukin (IL)-1β), C-X-C motif chemokine ligand-2 (CXCL-2), interferon (IFN)-γ, and IFN stimulated genes [MX dynamin-like GTPase-1 (MX1), 2’-5’-oligoadenylate synthetase-2 (OAS2)] by qPCR. Our data showed that SARS-CoV-2 exposure elicited an innate inflammatory/ antiviral response in different ocular tissues as evidenced by the induced expression of PRRs: TLR3, MDA5, inflammatory cytokines/chemokines: TNF-α, IL-1β, CXCL-2, and antiviral genes (IFNγ, MX1, and OAS2) (**Fig 2**). Moreover, in comparison with OC-exposed groups, the IN-exposures triggered remarkably higher inflammatory (TNF-α) and antiviral (IFN-γ, MX1, and OAS2) responses in retinal tissue. The innate response of the anterior segment tissue was mild and comparable in both IN and OC groups. The majority of these genes demonstrated a peak transcriptional response at 14 DPI in both AS and retinal tissue. These results indicated that IN exposure of SARS-CoV-2 prompted a hyperinflammatory immune response in the retina.

### SARS-CoV-2 permissively infects the cells lining the BRB, and hyperglycemia enhances their susceptibility

**Fig 3:**
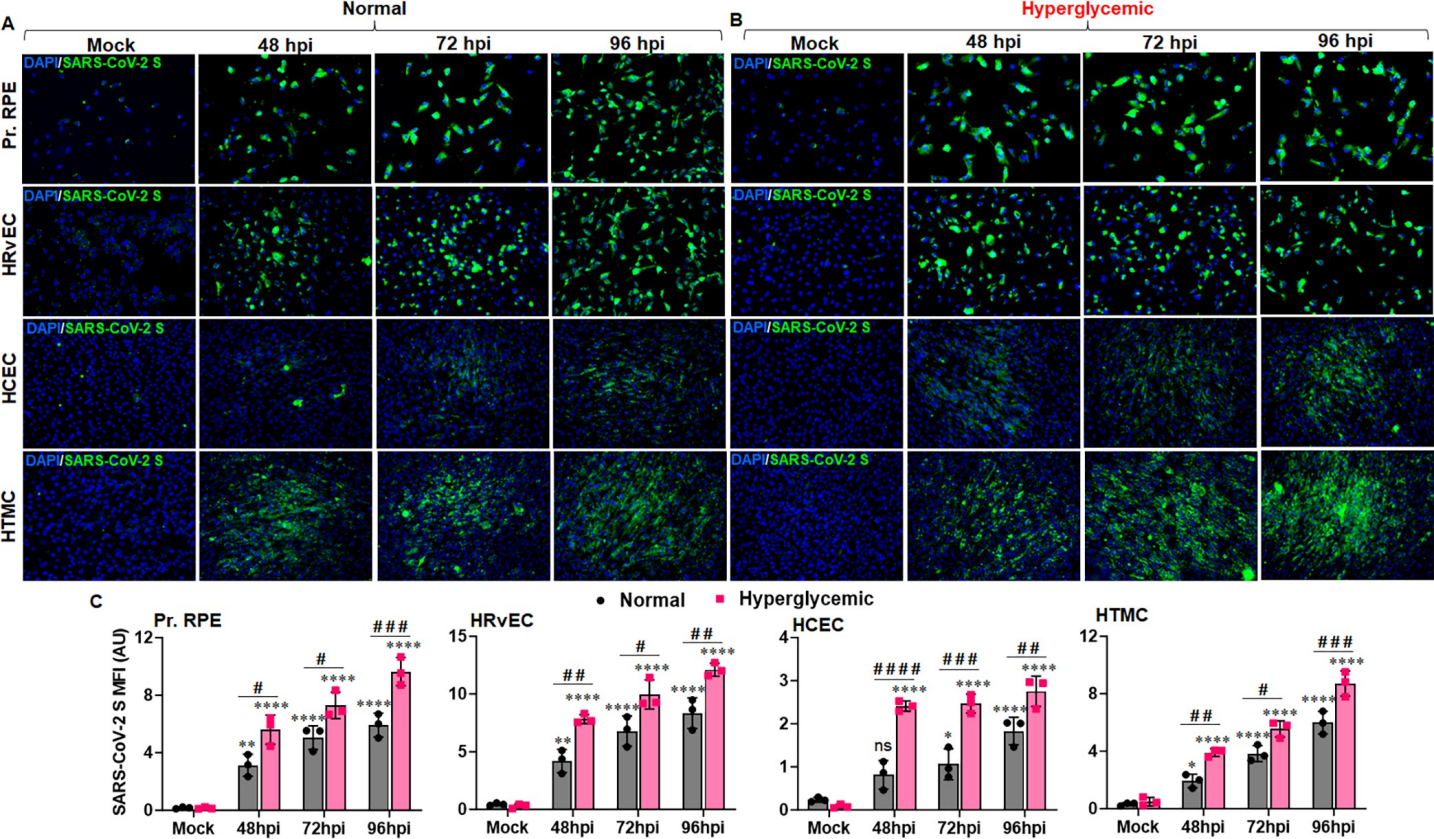
Cells lining the BRB are highly permissive to SARS-CoV-2 infection, and hyperglycemia enhances their susceptibility. Cells lining the outer BRB (Pr. RPE), inner BRB (HRvEC), corneal (HCEC), and TM (HTMC) cells were infected with SARS-CoV-2 strain USA-WA1/2020 at MOI:1 in **(A)** normal (5mM glucose) and **(B)** hyperglycemic (25mM glucose) conditions for indicated time points. Cells were fixed and stained for SARS-CoV-2 spike protein (Green) and DAPI nuclear stain (Blue). Image magnification, X 200. **(C)** The mean fluorescence intensity (MFI) for viral S antigen staining was quantified using ImageJ. The data represent mean ± SD of three biological replicates; * mock vs. SARS-CoV-2, # normal vs. hyperglycemic, # P< 0.05, **, ## P<0.005, ***, ### P<0.005, ****, #### P<0.0001, ns: not significant, two-way ANOVA.

In our mouse model, we observed IN exposure of SARS-CoV-2 disseminated the virus to various ocular tissues and induced a hyperinflammatory response in the retina, indicating a systemic permeation of the virus. The eye is an immune-privileged organ that is protected by a blood-retinal barrier; however, many infectious agents have been shown to breach the BRB and cause ocular complications. Based on our *in vivo* findings, we hypothesize that cells lining the BRB are the first to encounter SARS-CoV-2 for systemic exposure and can be breached by this virus. To test this hypothesis, we infected the cells lining the BRB, the outer BRB, retinal pigmented epithelium (RPE), and the inner BRB, retinal vascular endothelium (HRvEC) *in vitro*. SARS-CoV-2 permissively infected and replicated in primary human RPE and HRvEC cells, as evidenced by a time-dependent increase in the S-antigen positivity via IFA staining (**Fig 3A and 3C**). Surprisingly, we found primary human corneal epithelial cells (HCEC) comparatively resistant to SARS-CoV-2 infection (**Fig 3A and 3C**). SARS-CoV-2 has also demonstrated tropism towards the anterior segment of the eye, causing uveitis and glaucoma; therefore, we tested its infectivity towards human trabecular meshwork cells (HTMCs). Our results showed that HTMCs are susceptible to SARS-CoV-2 infection (**Fig 3A and 3C**). COVID-19 has demonstrated worse clinical outcomes in patients with comorbidities, such as diabetes. COVID-19 has been shown to induce hyperglycemia even in non-diabetic patients, which causes ACE2 glycation [44] and results in increased infectivity of hepatocytes [26]. To validate the BRB permissivity in comorbid conditions, we acclimatized BRB cells to normal and hyperglycemic conditions prior to infection with SARS-CoV-2. Our data indicate that hyperglycemia enhances the RPE, HRvEC, and HTMC susceptibility towards SARS-CoV-2 infection, whereas HCEC seemed less permissive to SARS-CoV-2 in these conditions (**Fig 3B and 3C**). In contrast to mock-treated cells, we observed a time-dependent SARS-CoV-2-induced cytopathy in RPE and HRvEC cells. Together, these findings confirm that BRB cells are highly prone to SARS-CoV-2 infection, and comorbid conditions can enhance BRB susceptibilities toward infection.

### SARS-CoV-2 induces the expression of viral entry receptors in BRB cells, and hyperglycemia potentiates their expression

**Fig 4:**
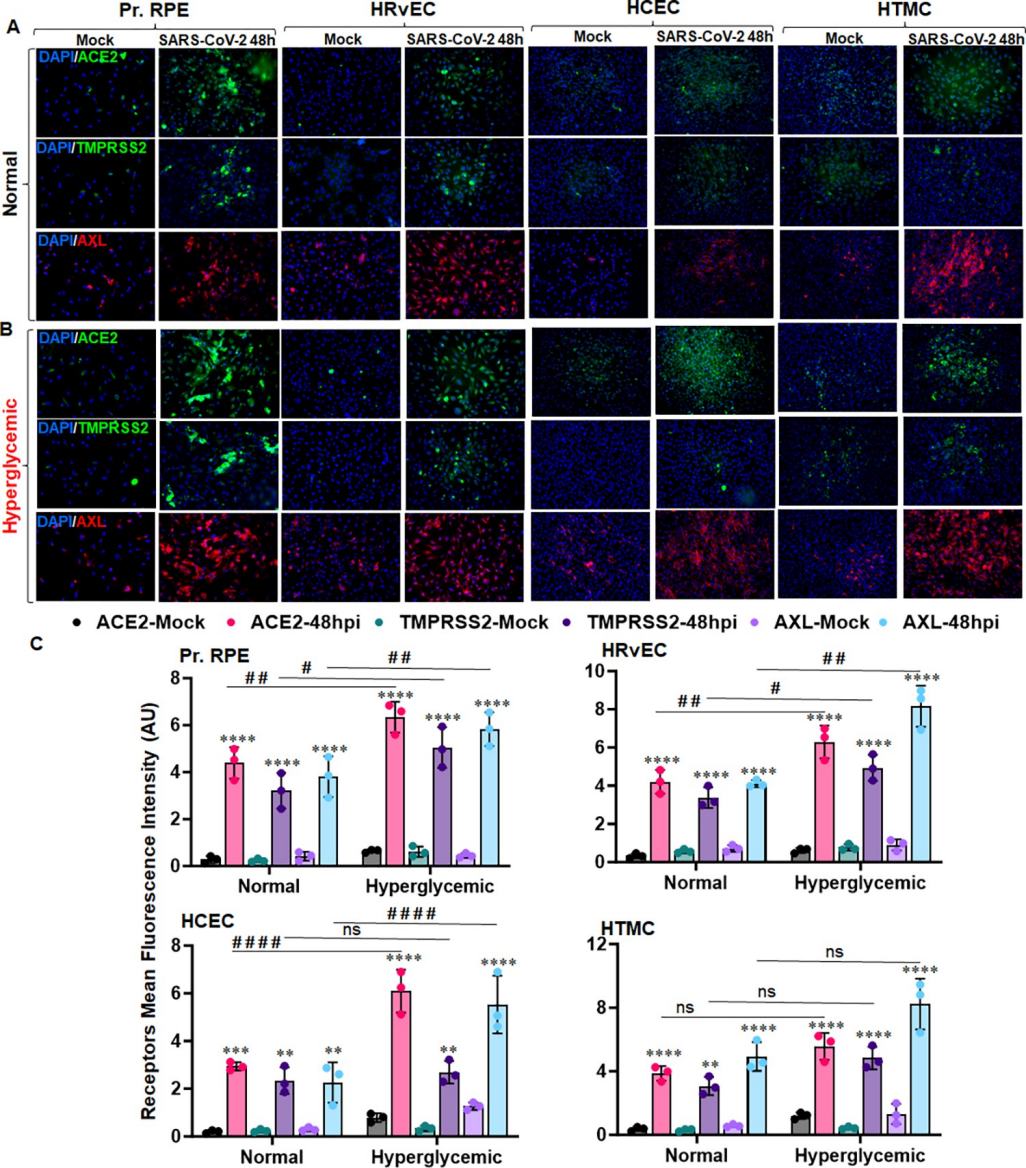
SARS-CoV-2 induces the expression of viral entry receptors in cells lining the BRB, and hyperglycemia potentiates their expression. Pr. RPE, HRvEC, HCEC, and HTMC cells were cultured in **(A)** normal (5mM D-glucose) and **(B)** hyperglycemic (25mM D-glucose) conditions and infected with SARS-CoV-2 strain USA-WA1/2020 at MOI:1 for 48h. Cells were fixed and subjected to SARS-CoV-2 entry receptors: ACE2 (green), TMPRSS2 (green), AXL (red), and DAPI nuclear stain (blue). Image magnification, X 200. **(C)** The mean fluorescence intensity (MFI) for viral entry receptors (ACE2, TMPRSS2, and AXL) staining was quantified using ImageJ. The data represent mean ± SD of three biological replicates. * mock vs. SARS-CoV-2, # normal vs. hyperglycemic, # P< 0.05, **, ## P<0.005, *** P<0.005, ****, #### P<0.0001, ns: not significant, two-way ANOVA.

Several viral entry receptors, such as ACE2, TMPRSS2, and AXL, have been implicated in SARS-CoV-2 entry in various cell types, including the ocular cells. To test the role of viral entry receptors in SARS-CoV-2 invasion in the eye, we assessed their expression in inner and outer BRB cells- RPE and HRvEC, along with HCEC and HTMC. Compared with the mock control, the SARS-CoV-2 challenge enhanced the expression of all three candidate receptors, ACE2, TMPRSS2, and AXL, in these cells, as evidenced by increased fluorescence positivity for these receptors in infected groups (**Fig 4A and 4C**). COVID-19 has been shown to modulate hyperglycemia and ACE2 expression, leading to increased complications in affected individuals[44]. Here, our results show that hyperglycemia augments the expression of ACE2, TMPRSS2, and AXL receptors on BRB cells upon infection (**Fig 4B and 4C**). These findings suggest that BRB cells express the SARS-CoV-2 entry receptors, and the virus may exploit ACE2, TMPRSS2, or AXL receptors for entry into the eye via BRB.

### SARS-CoV-2 elicits innate antiviral responses in BRB cells

**Fig 5:**
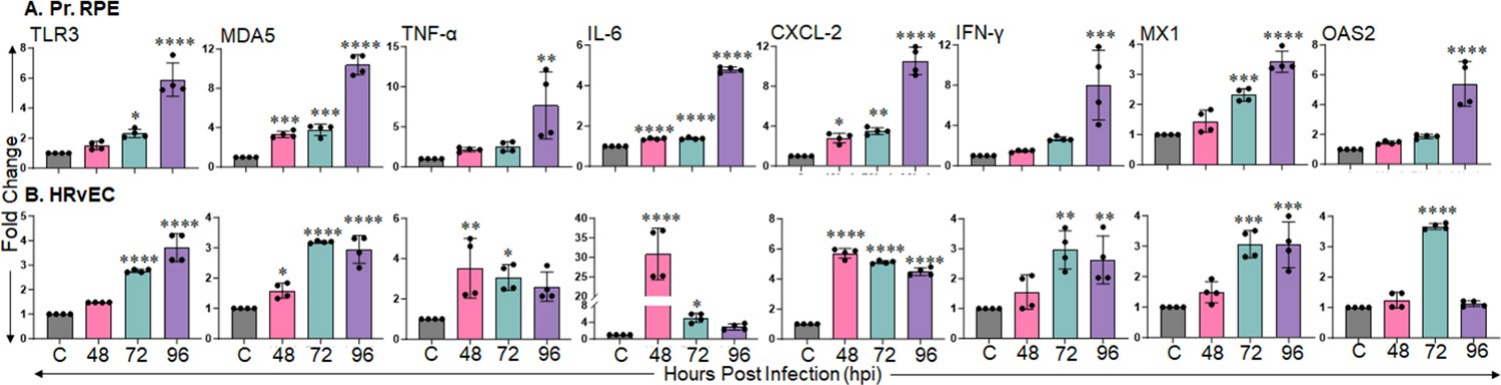
SARS-CoV-2 induces an innate antiviral immune response in cells lining the BRB. **(A)** Pr. RPE and **(B)** HRvEC cells were infected with SARS-CoV-2 strain USA-WA1/2020 at MOI:1 and collected at the indicated time points. Cells were harvested and subjected to RNA extraction and qPCR for indicated innate immune response genes. The data represent mean ± SD from four biological replicates. Statistically significant differences between the experimental groups were determined using one-way ANOVA. * P<0.05, ** P<0.005, *** P<0.005, **** P<0.0001.

As SARS-CoV-2 demonstrated the induction of multiple entry receptors and established a permissive infection in BRB cells, we next sought to determine the innate immune response triggered by these cells upon infection. We infected primary human RPE and HRvEC cells with SARS-CoV-2 for 48, 72, and 96 h and measured the expression of PRRs: TLR3, MDA5, inflammatory cytokines/chemokines: TNF-α, IL-6, CXCL-2, and antiviral genes (IFNγ, MX1, and OAS2) by qPCR. Our data showed that SARS-CoV-2 infection significantly induced the expression of TLR3, MDA5, TNF-α, IL-6, CXCL-2, IFNγ, MX1, and OAS2 genes in comparison to uninfected control in both RPE (**Fig 5A**) and HRvEC cells (**Fig 5B**). Most of these genes showed a time-dependent induction upon SARS-CoV-2 infection in BRB cells, indicating virus-induced immune alterations.

### SARS-CoV-2 induces BRB cell death, which is further augmented by hyperglycemia

**Fig 6:**
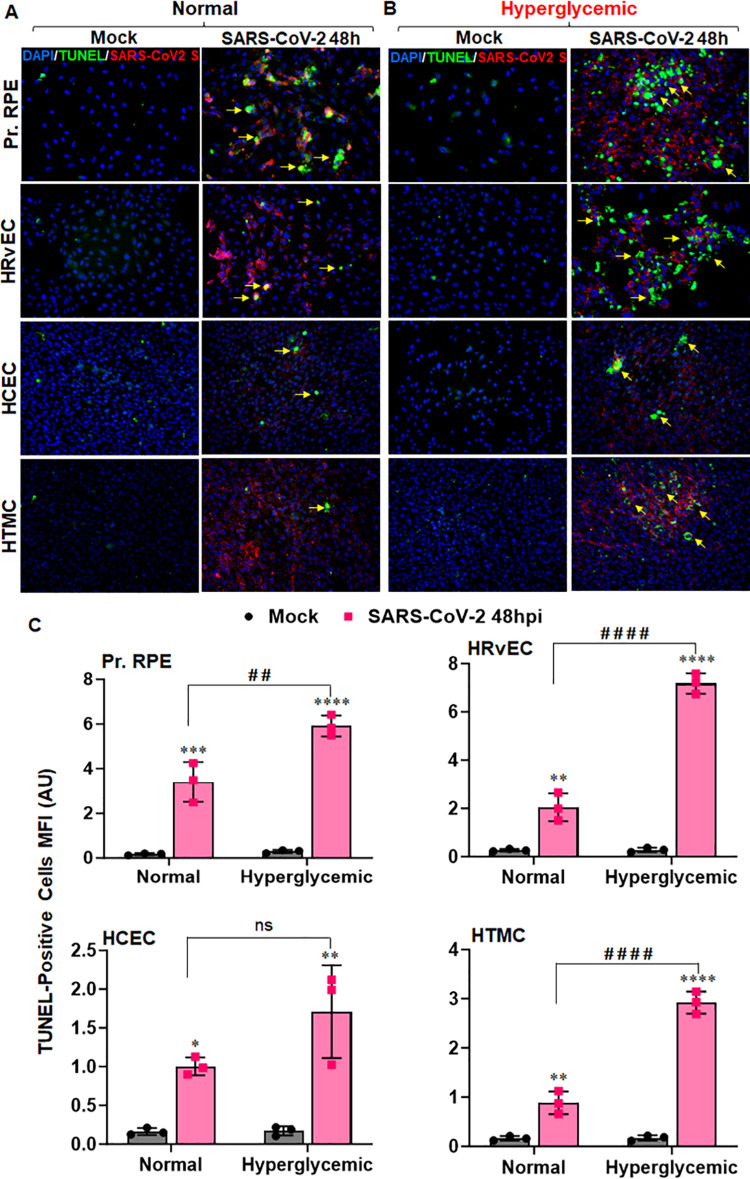
SARS-CoV-2 induces BRB cell death that is aggravated by hyperglycemic conditions. Pr. RPE, HRvEC, HCEC, and HTMC cells were cultured in **(A)** normal (5mM D-glucose) and **(B)** hyperglycemic (25mM D-glucose) conditions and infected with SARS-CoV-2 strain USA-WA1/2020 at MOI:1 for 48h. Cells were fixed and subjected to TUNEL assay (Green- a few representatives indicated with yellow arrow) with immunostaining for SARS-CoV-2 Spike (Red) and DAPI nuclear stain (Blue). Image magnification, X 200. **(C)** The mean fluorescence intensity (MFI) for TUNEL-positive cells was quantified using ImageJ. The data represent mean ± SD of three biological replicates. * mock vs. SARS-CoV-2, # normal vs. hyperglycemic, * P< 0.05, **, ## P<0.005, *** P<0.005, ****, #### P<0.0001, ns: not significant, two-way ANOVA.

Cell death mechanisms play a vital role in maintaining cellular homeostasis and normal cell functionality. Besides triggering inflammatory immune response, many viruses can initiate programmed cell death in infected cells. SARS-CoV-2 has been shown to induce cell death of different cell types that exert complicated effects on the antiviral immunity of the host [45]. Because cells lining the BRB showed high permissivity towards SARS-CoV-2, we next determined whether SARS-CoV-2 induces cell death in these ocular cells. We performed a TUNEL assay to measure the cell death. Our results show that SARS-CoV-2 induces cell death in RPE and HRvEC cells, as evidenced by the increased TUNEL-positive cells in comparison to uninfected controls (**Fig 6A**). Hyperglycemia further enhanced the virus-induced cell death in these ocular cells (**Fig 6B**). HCEC and HTMC cells were found to be comparatively resistant to SARS-CoV-2-induced cell death, which may be attributed to their reduced permissivity. These results demonstrate that SARS-CoV-2 infection can induce cell death in BRB, which may help disseminate the virus in addition to pathological consequences.

### Long-term exposure of SARS-CoV-2 spike antigen can cause retinal pathologies

**Fig 7:**
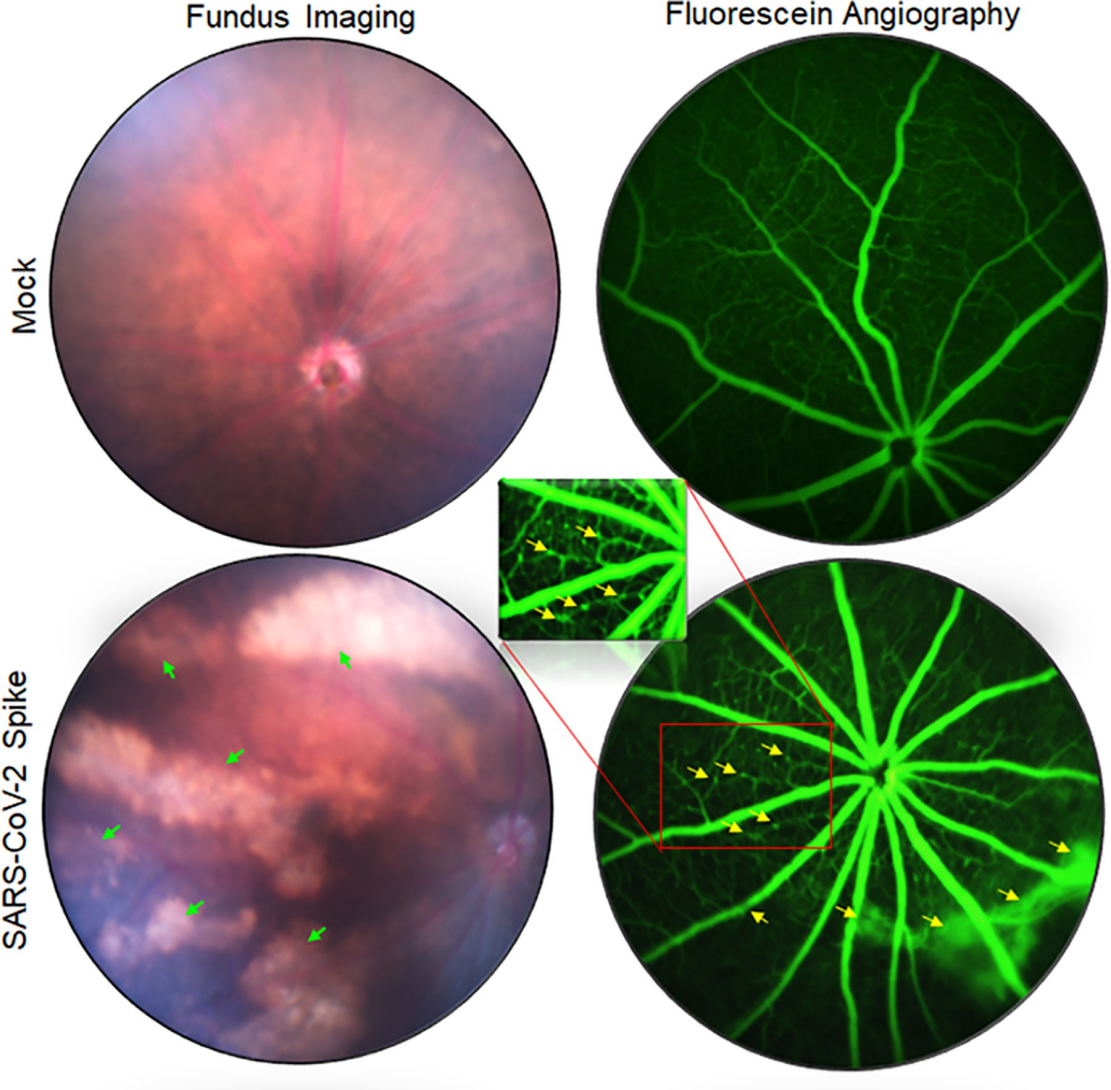
SARS-CoV-2 spike protein causes retinal atrophy, microaneurysm, vein occlusion, and vascular leakage in the eye. C57BL/6J mice (n = 5) were intravitreally injected with SARS-CoV-2 Spike protein (100ng/eye). Thirty days post-injection, eyes were imaged using Micron IV fundus imager. The representative fundus image and fluorescein angiography show retinal atrophy (indicated with green arrows), microaneurysm, vein occlusion, and vascular leakage (marked with yellow arrows) in the eyes.

The long-term effects of COVID-19 on visual function and ocular anatomy are not yet fully understood. Some clinical studies have shown that SARS-CoV-2 can cause retinal vascular occlusions, including central retinal vein occlusion (CRVO), central retinal artery occlusion (CRAO), vitritis, and acute retinal necrosis (ARN) [46]. Because we observed the presence of S-antigen in various parts of the eye and SARS-CoV-2-induced cell death in cells lining the BRB, we aimed to evaluate the long-term consequences of S-antigen on overall retinal health. To study this, we intravitreally injected recombinant S-protein in C57BL/6J (B6) mouse eyes and assessed the retinal pathology 30 days post-injection using fundus imaging and fluorescence angiography. Our results showed that S-protein caused microaneurysms, retinal atrophy, RPE mottling, and vein occlusion in the retina in contrast to the mock-treated animals (**Fig 7**). These findings indicate that the extended presence of viral S-protein can cause retinal vascular changes and long-term pathologies in exposed eyes, which may equate with long-COVID sequelae.

## Discussion

COVID-19 is an acute respiratory disease where severe lung inflammation is the primary clinical manifestation. In addition to causing respiratory illness, several ocular manifestations have been reported in COVID-19 patients. However, the ability of SARS-CoV-2 to invade ocular tissue and its effects on ocular health remains unclear. With recent clinical findings, it is becoming increasingly clear that SARS-CoV-2 infection has broad implications in ocular disease sequelae [4,5,15]. The ocular mucosal milieu has been identified as the most susceptible region that can be affected by transmissible microorganisms, including viral and bacterial pathogens such as herpes simplex virus-1, influenza, Zika, and *Staphylococcus aureus* [16,28,47–51]. Previous studies have shown the presence of SARS-CoV-2 RNA and proteins in various ocular tissues and fluids, including conjunctiva, limbus, tears, and aqueous humor, indicating that the eye could be a potential site of infection and transmission [21,25,38]. It is believed that SARS-CoV-2 can be disseminated through the ocular surface via nasolacrimal ducts, which connect the ocular surface with the respiratory tract, facilitate aqueous exchange, and provide shared lymphoid tissues between these sites [50,51]. Although we did not observe the spreading of the virus to systemic organs via ocular surface exposure, further studies on viral spread through the nasolacrimal duct are warranted.

The BRB is an essential component of ocular health and, much like the blood-brain barrier (BBB), allows metabolic homeostasis while maintaining the immune privilege of the eye. The BRB is composed of an outer barrier of retinal pigment epithelium (RPE) and an inner barrier of retinal vascular endothelium (RvEC). In the current study, we found that both of these BRB cell types were highly permissive for SARS-CoV-2 infection *in vitro*. This is consistent with our observations that the IN challenge of SARS-CoV-2 in K18-hACE2 mice led to viral positivity in different parts of the eye, including the retina and the anterior segment tissue in addition to high viral titers in the lungs and brain. Our study is consistent with previous findings where SARS-CoV-2 has shown tropism and high titers in various distal organs such as lungs, brain, trigeminal nerve, and optic nerve following intranasal challenge in K18-hACE2 mice [39,52]. Moreover, we observed that IN exposure induced a robust inflammatory response in the retina, indicating a systemic permeation of the virus. Notably, nasal epithelia have been shown to support SARS-CoV-2 replication with delayed antiviral response and may transmit the virus upward to the eye following intranasal exposure [53,54]. These findings indicate SARS-CoV-2 can cross both the BRB and the BBB via systemic exposure, which suggests there may be a conserved mechanism for ocular- and neuroinvasion by the virus. Conversely, in our study, ocular exposure led to viral positivity in the eye, it did not cause widespread viremia or mortality in K18-hACE2 mice. We observed viral RNA and S-protein positivity in different organs till 21 days, with a peak at 14DPI. Our findings are consistent with some clinical reports showing ocular manifestation in patients varied from 15 days to two months post-infection [55,56]. Moreover, a study has demonstrated the presence of viral RNA in ocular swabs from a COVID-19 patient with ocular manifestations by day 21–27 from the onset of symptoms [57]. We observed that HTMCs were permissive to SARS-CoV-2 infection, indicating its tropism towards the anterior segment, whereas the corneal epithelial cells were relatively resistant, suggesting that the cornea may serve as a physical barrier that prevented SARS-CoV-2 systemic spread through the ocular surface exposure. Our findings corroborated a recent study showing that ocular exposure fails to transmit the virus to the lungs in K18 mice as well as Syrian hamsters [47]. This study demonstrated SARS-CoV-2 ocular tropism through trigeminal and optic nerves. Several candidate receptors, including ACE2, TMPRSS2, and AXL, have been implied in SARS-CoV-2 entry in different cell types [58]. Many ocular cells, including cornea, conjunctiva, and RPE, have been shown to express these receptors [19,59]. In our study, we observed the induction of these entry receptors upon exposure to the virus despite differential susceptibilities to ocular cell types, indicating that other factors associated with the viral replication cycle or host antiviral responses may be ultimately responsible for this observation. Together, our observations imply that the retina is highly susceptible to SARS-CoV-2 infection via a breach in the BRB. This is consistent with studies of other viruses that breach the BRB and cause ocular manifestations, including Zika and Ebola [47,60]. The molecular mechanism behind the breakdown of BRB is likely multifactorial. SARS-CoV-2 has been shown to exploit host machinery to create a permissive host microenvironment by modulating immune response, various phases of cell cycle, and cell death pathways by its structural, non-structural, and accessory proteins. SARS-CoV-2 encodes 4 structural, 16 non-structural, and multiple accessory proteins, and many of them have been implicated in tempering with host cell signaling [61]. Notably, SARS-CoV-2 NSP1, NSP15, ORF6, ORF7a, ORF7b, and ORF8 are involved in suppressing host IFN responses, while NSP2 and NSP3 antagonize host immune response via facilitating viral genome assembly and disrupting the cell cycle progression and apoptosis[61]. ORF3a is identified as an ion channel and is known to block autolysosome maturation, whereas NSP6 is shown to restrict host autophagosome expansion and facilitate viral replication and spread [61,62]. In this study, we observed that in addition to the upregulation of receptors associated with SARS-CoV-2 cell entry and cytokine response, both BRB cell types had a significant amount of apoptotic cell death induced by viral infection. SARS-CoV-2 has been shown to induce cell death in several tissues, which significantly contribute to viral spread and pathogenicity. Induction of cell death programming is an innate immune mechanism that can facilitate the clearance of pathogens and infected cells; however, when it becomes unregulated or manipulated, it can result in tissue damage, prevent inflammatory homeostasis, and contribute to pathogenesis [45].

The induction of cell death programming is far from the only innate immune response initiated upon viral cell entry. SARS-CoV-2 induces a systemic cytokine storm in the lungs that, instead of leading to viral clearance, contributes to pathogenesis and can lead to ARDS, a primary cause of death in COVID-19 patients. Several proinflammatory mediators are associated with SARS-CoV-2 infection in multiple tissues, including the retina [39,55]. Consistent with this, we observed the induction of a robust immune and antiviral response in the retina during IN challenge. In contrast to IN exposure, OC exposure induced a mild inflammatory response in the ocular tissue. Furthermore, we observed the induction of several PRRs (TLR3, MDA5), proinflammatory mediators (TNF-α, IL-6, CXCL-2), and antiviral effectors (IFNγ, MX1, OAS2) genes in RPE and HRvEC cells. TLR3 and MDA5 are endosomal and cytosolic PRRs involved in sensing RNA viruses and activating downstream antiviral and inflammatory responses. A time-dependent increase in the expression of these PRRs following SARS-CoV-2 infection suggests activation of antiviral signaling to curb the pathogen spread in BRB cells, which is also evident with the heightened expression of TNF-α, IL-6, CXCL-2, and IFNγ alongside IFN stimulated genes (ISGs): MX1 and OAS2. While these cytokines, IFNs, and IGSs act together to restrict viral replication in the ideal situation, a dysregulated immune response may aggravate the tissue homeostasis and cause host-induced damage to immune privileged retina. Interestingly, we found persistent activation of these inflammatory mediators in K18-hACE2 mice retina as well as in cells lining the BRB *in vitro* upon SARS-CoV-2 infection. These findings are consistent with studies showing that SARS-CoV-2 can induce retinal inflammation in human patients [54] as well as mouse models [47].

Diabetes is recognized as a comorbidity associated with increased morbidity and mortality during SARS-CoV-2 infection. High expression of ACE2 in diabetics has been reported to cause severe infection in these patients [56]. Many clinical studies including meta-analyses have reported increased incidence of SARS-CoV-2 infectivity, disease severity, and mortality associated with COVID-19 in diabetics as compared to non-diabetics individuals [57]. Studies have indicated that preexisting or new onset hyperglycemia may be associated with a poor outcomes in individuals affected by COVID-19 [56,57]. There are several factors that may contribute to this increased risk. There is a general increase in susceptibility to infections associated with diabetes, likely due to impairment of the immune response due to chronic hyperglycemia. Hyperglycemia is known to induce endothelial dysfunction, compromising the integrity of blood vessels. Once compromised, retinal endothelial cells could create a permissive environment for SARS-CoV-2 to breach the barrier. This is consistent with our observations that hyperglycemic conditions were associated with increased SARS-CoV-2 infectivity in cells lining the BRB. Whether this is due to increased viral uptake, replication, or changes in antiviral responses is unclear, as it potentiated viral receptor expression and also led to increased BRB cell death. The mechanism underlying these observations may be related to the breakdown of the BRB during diabetes, though that is beyond the scope of this current study.

COVID-19 has demonstrated long-term consequences on human health, commonly referred to as long-COVID or post-acute sequelae of COVID (PASC). Contemporary studies also suggest that COVID-19 continues to pose significant risks to public health, and the rate of infection is directly or indirectly linked to the development of long-term implications that have an adverse effect on respiratory, ocular, and, most importantly, brain health [63,64]. SARS-CoV-2 has shown retinal vascular changes in COVID-19 patients, including CRVO, CRAO, ARN, and retinitis. However, the effect of long-COVID on ocular disease sequelae remains unknown. Here, we demonstrated the impact of the extended presence of S-antigen on retinal health, which may correlate to the adverse outcomes of sustained SARS-CoV-2 infection in affected individuals. Our data using a recombinant SARS-CoV-2 spike showed the induction of microaneurysms, RPE mottling, retinal atrophy, and retinal vein occlusion by S-protein in mouse eyes.

Despite the importance of several key findings, we acknowledge some limitations to our study. First, we used K18-hACE mice in this study; this mouse model has shown higher tropism towards CNS and lethal encephalitis, in addition to lung diseases that are not common in human patients [65]. In addition, the hACE2 transgene expression in these mice is driven by a non-native cytokeratin-18 promoter, that are distinct from endogenously expressed ACE2 and may not be physiologically relevant. The altered expression of ACE2 receptor in K18-hACE2 mice have shown heightened pathology with ancestral SARS-CoV-2 strain in comparison to immunocompetent WT mice. Therefore, some recent studies have developed mouse-adapted strains with N501Y substitution, also present in many of the SARS-CoV-2 variants that have shown infectivity in WT mice [65]. Studies comparing the ocular pathology in WT and K18-hACE2 mice using either mouse-adapted strains or other variants of SARS-CoV-2 are needed to discern the role of BRB in SARS-CoV-2 pathogenesis; however, that is beyond the scope of the current study. We also acknowledge the limitation of our study using a recombinant spike protein to evaluate long COVID-induced retinal pathology. Additional studies following the resolution of live viral infection are warranted to assess the long-term consequences of viral remnants on retinal health.

**Fig 8:**
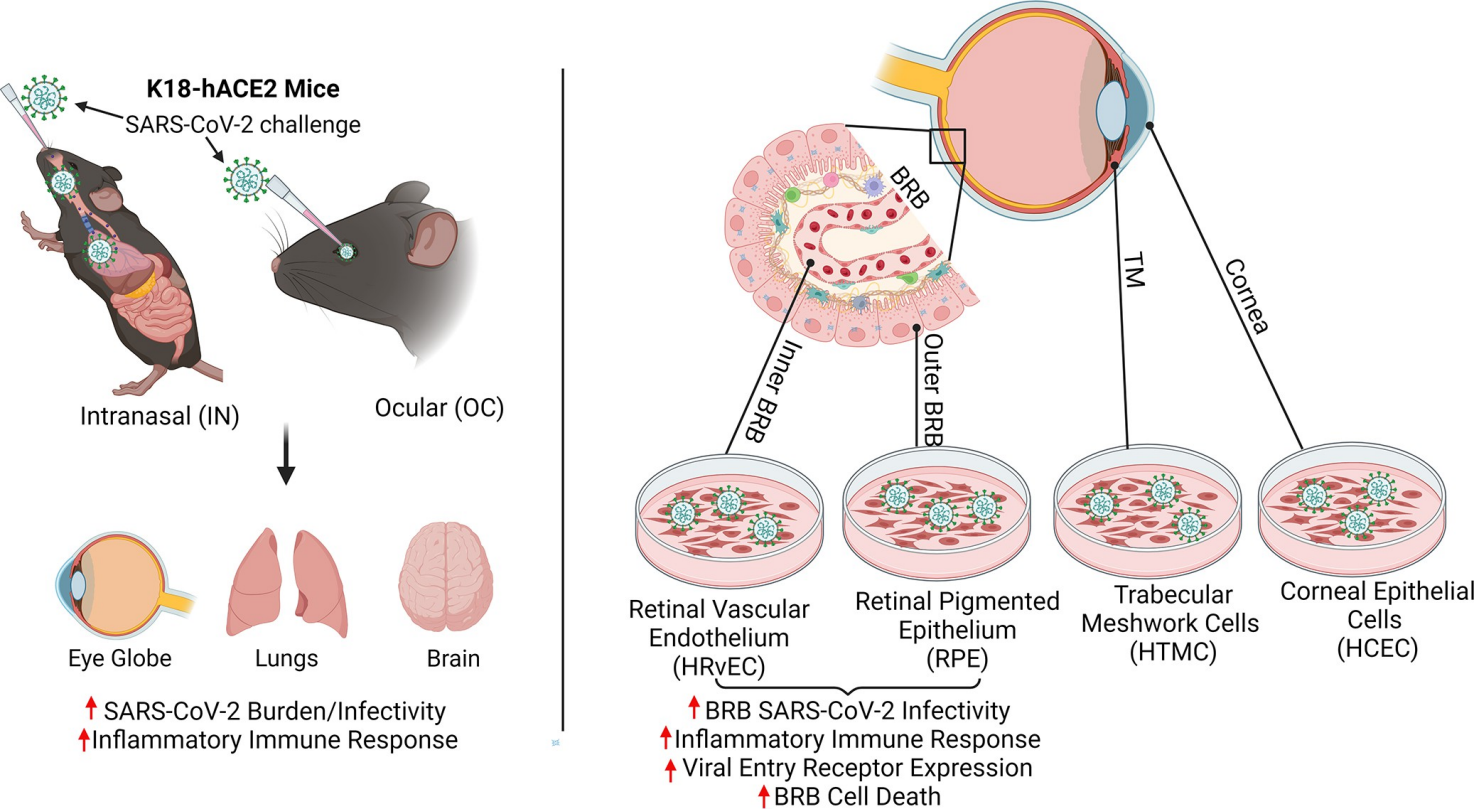
A schematic illustration of SARS-CoV-2 ocular tropism via cells lining the blood-retinal barrier (BRB). Intranasal exposure of SARS-CoV-2 causes ocular tropism and induction of hyperinflammatory response in the retina through BRB infection, whereas ocular exposure does not cause lung infection and moribund illnesses in K18-hACE2 mice despite the extended presence of viral remnants in various ocular tissues. Cells lining the BRB, outer BRB: RPE, and inner BRB: HRvEC are highly permissive to SARS-CoV-2 infection, whereas corneal epithelial cells are comparatively resistant to infection. The schematic diagram was created using BioRender software (BioRender.com).

In summary, our study demonstrated that the ocular infection by SARS-CoV-2 occurs during both OC and IN exposures. The IN route can disseminate the virus to the eye; in contrast, the OC exposure fails to transmit the virus to distal organs or cause lethal infections. The induction of hyperinflammatory retinal responses in IN-exposed groups and the increased permissivity of cells lining the BRB (RPE and HRvEC) indicates a systemic permeation of the virus through the BRB (**Fig 8**). BRB cells express receptors for viral entry and are highly prone to SARS-CoV-2-induced cell death. Moreover, our data demonstrated that the SARS-CoV-2 ocular manifestations are exacerbated by comorbidities. Further studies are required to assert the long-term consequences of SARS-CoV-2 live infection on retinal health. Conclusively, our study demonstrates the critical role of BRB in SARS-CoV-2 infection and ocular tropism, which will help in designing therapeutic strategies to treat or prevent COVID-19-mediated ocular complications.

## Methods

### Ethics statement

The SARS-CoV-2 animal experiments were conducted at the University of Missouri, Laboratory for Infectious Disease Research (LIDR), BSL3 facility. All mice were treated in compliance with the Association for Research in Vision and Ophthalmology (ARVO) Statement for the Use of Animals in Ophthalmic and Vision Research. The biohazard and animal procedures were approved by the University of Missouri Institutional Biosafety Committee (IBC) and the Animal Care and Use Committee (ACUC) under protocol # 11160 and 43226 respectively.

### Cells and culture conditions

Primary human retinal pigmented epithelial (RPE) [47] cells were maintained in RtEGM Bulletkit medium (Lonza, Walkersville, MD). Primary human retinal microvascular endothelial cells (HRvEC) [66] were cultured in EGM-Plus Endothelial Cell Growth Media-Plus BulletKit (Lonza, Lonza, Walkersville, MD). Primary human corneal epithelial cells (HCEC) [67] were grown in a corneal epithelial cell basal medium supplemented with corneal epithelial cell growth kit (ATCC, Manassas, VA). Primary human trabecular meshwork cells (HTMC) [68] were cultured in Dulbecco’s modified Eagle’s medium (DMEM) supplemented with 10% fetal bovine serum (FBS), 10 μg/mL L-glutamine, and 1X penicillin-streptomycin solution (ThermoFisher Scientific, Rockford, IL). *Cercopithecus aethiops* kidney cells (Vero E6; ATCC CRL-1586) were cultured in Eagle’s Minimum Essential Medium containing Earle’s Balanced Salt Solution, non-essential amino acids, 10 μg/mL L-glutamine, and 1X penicillin-streptomycin supplemented with 2% FBS. For hyperglycemia studies, cells were adapted to high (25mM) and normal (5mM) D-glucose-containing media as described previously [69].

### Mice

K18-hACE2 (B6.Cg-Tg(K18-ACE2)2Prlmn/J) mice were purchased from Jackson Laboratory (Bar Harbor, ME) and bred in-house at the University of Missouri Mutant Mouse Resource & Research Center (MMRRC). C57BL/6J (B6) wild-type (WT) mice were purchased from Jackson Laboratory (Bar Harbor, ME) and maintained at the University of Missouri School of Medicine Office of Animal Resources (OAR) facility. Both male and female mice aged 6–10 weeks were used for all experiments. All animals were housed in a controlled-access, The Association for Assessment and Accreditation of Laboratory Animal Care (AAALAC) approved, OAR facility, maintained in a 12:12 h light/ dark cycle, and fed on lab diet rodent chow (Labdiet Pico Laboratory, Saint Louis, MO) and water *ad libitum*.

### SARS-CoV-2 Strain and infection procedure

SARS-CoV-2 strain USA-WA1/2020 was obtained from BEI resources, NIAID, NIH, and propagated at low passage in the ATCC CRL-1586 Vero E6 cell line. The titers were determined by plaque assay or viral RNA genomic equivalent (GE) copy number counts using quantitative PCR. For *in vitro* studies, primary human RPE, HRVEC, HCEC, and HTMC were infected with SARS-CoV-2 at MOI-1. Uninfected cells were used as a mock control. For *in vivo* studies, K18-hACE2 transgenic mice were challenged with SARS-CoV-2 (0.4x10^3^ PFUs in PBS) either by ocular [OC-as an eye drop (5μL) on both eyes] or intranasal (IN-drop inoculation (15μL/naris, 30μL total) routes under anesthesia. Mice treated with PBS were used as mock controls. SARS-CoV-2-challenged mice were monitored daily for symptoms of infection, including weight loss and lethargy. Moribund mice were euthanized. At respective endpoints, mice were euthanized, and lungs, brains, and eyes were sterilely retrieved. To inactivate SARS-CoV-2, organs were placed in 10% neutral buffered formalin or TRIzol prior to further analysis. For extended spike (S) protein challenge studies, C57BL/6 WT mice were intravitreally injected with 0.1μg of S-protein. Thirty days post injections, funduscopic examination and fluorescein angiography were performed under anesthesia using Micron IV (Phoenix Research Lab, Pleasanton, CA).

### Immunofluorescence staining

For immunostaining (IFA) procedures, cells were cultured on a Nunc four-well chamber slide (Fisher Scientific, Rochester, NY) and infected with SARS-CoV-2 at MOI of 1. At 48-, 72-, and 96-hours post-infection (hpi), infected and mock-treated cells were fixed with 4% paraformaldehyde in PBS at 4°C. For *in vivo* samples, 10μM thick eye, lungs, and brain cryosections were fixed in 4% paraformaldehyde, as indicated above. After three PBS washes, the cells and tissue sections were blocked and permeabilized using 1% (w/v) BSA with 0.4% Triton X-100 or 10% (w/v) goat serum containing 0.4% Triton X-100 made in PBS for 1 h at room temperature. The cells/tissue sections were incubated with primary mouse/rabbit monoclonal antibodies, anti-SARS-CoV spike (S) (BEI resources catalog NR-616), AXL (Cell Signaling, catalog 8661S), ACE2 (Santa Cruz Biotechnology, catalog sc-390851), or TMPRSS2 (Santa Cruz Biotechnology, catalog sc-515727) antibodies (1:100 dilution) overnight at 4°C. After removal of the primary antibodies, the cells/tissue sections were washed extensively with PBS and incubated with anti-mouse/rabbit Alexa Fluor 488/594-conjugated secondary antibody (1:200) for 1 hour at room temperature. Following incubation, cells/tissue sections were extensively washed with PBS and mounted in Vectashield anti-fade mounting medium containing DAPI (Vector Laboratories, Burlingame, CA). The slides were imaged using a Keyence microscope (Keyence, Itasca, IL).

### RNA Extraction and Quantitative RT-PCR

Total RNA was extracted from SARS-CoV-2 infected and mock-treated cells and tissues using TRIzol reagent per manufacturer’s instructions (Thermo Scientific, Rockford, IL). cDNA was synthesized using 1 μg of total RNA using a Maxima first-strand cDNA synthesis kit, per the manufacturer’s instructions (Thermo Scientific, Rockford, IL). The cDNA was amplified using gene-specific PCR primers using QuantStudio 3 Real-Time PCR system (ThermoFisher Scientific, Rockford, IL). The relative expression of these genes were normalized in proportion to the constitutive gene 18s RNA as an internal control and quantitatively analyzed using the ΔΔC_T_ method and represented as fold change expression. The viral RNA was amplified using Applied Biosystems TaqMan Fast Virus 1-Step Master Mix (Thermo Scientific, Rockford, IL) and SARS-CoV-2 Nucleocapsid (N) gene-specific TaqMan probes (Integrated DNA Technologies, Coralville, IA).

### Terminal dUTP Nick End-Labeling (TUNEL) Assay

SARS-CoV-2-induced cell death was evaluated using a TUNEL assay. Cells were grown in normal (5mM-glucose) and hyperglycemic (25mM-glucose) conditions and challenged with SARS-CoV-2 (MOI of 1) in Nunc four-well chamber slides (Fisher Scientific, Rochester, NY) for the indicated time points. TUNEL staining was performed using ApopTag Fluorescein In Situ Apoptosis Detection Kit per the manufacturer’s instructions (Millipore, Billerica, MA). The TUNEL-stained cells were visualized using a Keyence microscope (Keyence, Itasca, IL).

### Statistical analysis

The statistical differences between experimental groups were analyzed using GraphPad Prism10 (GraphPad Software, La Jolla, CA). The one-way analysis of variance (ANOVA) and two-way ANOVA were used to compare the significance level between experimental groups, as indicated in figure legends. A value of *P*<0.05 was considered statistically significant. All data are expressed as the means ± standard deviation (SD) from three or four biological replicates unless indicated otherwise.
